# Emulsification of Silicone Oils: Altering Factors and Possible Complications—A Narrative Review

**DOI:** 10.3390/jcm13082407

**Published:** 2024-04-20

**Authors:** Małgorzata Łątkowska, Małgorzata Gajdzis, Radosław Kaczmarek

**Affiliations:** Department of Ophthalmology, Wrocław Medical University, 50-556 Wrocław, Poland

**Keywords:** silicone oil, emulsification, heavy silicone oils, endotamponade, OCT

## Abstract

**Background**: Endotamponade of the vitreous body with silicone oil is a common procedure, being the basis of many vitreoretinal surgeries. However, emulsification may happen, which is a clinically relevant adverse event of silicone oil use. **Methods**: This review provides a thorough analysis of the emulsification process. It focuses on describing factors affecting this event as well as its possible subsequent complications. **Results**: The viscosity of silicone oil, the duration of emulsification, the status of the lens and many other factors have an influence on the onset and intensity of emulsification. This phenomenon carries several risks for operated eyes such as increased intraocular pressure, keratopathy or structural changes to the retina. **Conclusions**: The use of modern imaging techniques, especially optical coherence tomography, enables faster detection of the emulsification process. This allows for an adequate clinical response and more accurate follow-up of the patient.

## 1. Introduction

The process of emulsification is a clinically important complication of the use of silicone oil in vitrectomy procedures and is one of the most serious potential problems faced by patients with silicone oil (SO)-filled eyes. This phenomenon involves precipitation of oil droplets over time. Time-dependent breakdown of the oil into smaller and smaller droplets causes the viscoelastic to lose its cohesiveness. The condition that favors the initiation of this process is a decrease in surface tension. It occurs in the presence of surfactants, which reduce tension between two phases. Endogenous molecules with such properties include proteins, phospholipids and lipoproteins [1,2].

It can be difficult to reliably assess the occurrence of emulsification due to the very small size of the oil particles. Most reports of this phenomenon only refer to the presence of SO emulsification without an objective assessment of its progression. The diagnosis of emulsification is usually clinical—the size and quantity of oil droplets are not specified. Often the only diagnostic test performed is a slit lamp examination or gonioscopy. As noted by Chan et al., most droplets with sizes smaller than 2 μm are not detectable. Therefore, it is suspected that the presence of SO emulsification is underestimated [3]. Currently, thanks to newer examination techniques such as OCT, this adverse event can be detected with greater accuracy.

To fully understand the emulsification process, it is crucial to learn the chemical and physical properties of silicone oils. To this end, studies have been conducted to find out what factors may affect the earlier onset or intensity of this process and what consequences it may have on patients.

## 2. Silicone Oil as a First-Choice Tamponade and Its Characteristics

### 2.1. Wide Clinical Application of SO

Silicone oil has been used in ophthalmic surgery for many years, starting in the 1960s [4]. It is used to tamponade the vitreous chamber. Over the years it has progressively gained importance in retinal surgery due to its desirable physical and chemical properties. It is commonly used in vitrectomies in patients with rhegmatogenous retinal detachment, retinal detachment with proliferative vitreoretinopathy, proliferative diabetic retinopathy and ocular trauma [5,6,7]. Compared with gas, it provides faster visual acuity rehabilitation and greater independence regarding the recommended posttreatment position for the patient, which is especially important in cases of noncompliance. Moreover, in many cases a longer tamponade time is desirable [8]. Nevertheless, for years concerns have been raised regarding potential toxicity and complications. Here we describe its characteristic properties that give oil such a wide range of applications.

### 2.2. Physicochemical SO Properties

In chemical terms silicone oil is a synthetic polymerized liquid, which consists of repeating siloxane with an organic chain. There are different types of silicone oils with different molecular weights, chain lengths or organic residues, which correspondingly change their properties. In ophthalmology, silicone oils with hydrocarbon radicals as side groups are mainly used. The principal type of polymer is polydimethylsiloxane (PDMS). It is optically transparent; hence, it can serve as a substitute for the vitreous body. The solid form of SO is used for scleral buckling [9,10].

A unique property that determines its vitrectomy application is high interfacial tension between oil and water, which is the force between their surfaces. It prevents leakage of vitreous humor through tears in the retina into the subretinal space. This facilitates effective coverage of breaks and provides appropriate retinal tamponade [11]. Another characteristic of SO is buoyancy. It is an upward force that counteracts the force of gravity. Both these factors act on oil bubbles. Standard SO has an average specific gravity of ≈0.97—a value lower than water and vitreous humor, which means that SO floats in the vitreous cavity. However, it is believed that buoyancy of bubbles does not significantly affect the closure of retinal tears, unlike the above-mentioned interfacial tension. Moreover, if we look at the alternative endotamponades, i.e., gases, oils have a low buoyancy compared to them [10,11,12].

The most important property of oils in surgical terms is viscosity. It means resistance to deformation at a certain rate, expressed in centistokes (1 cSt = 10^−6^ m^2^/s). The final SO product is actually a mixture of PDMS with different chain lengths. Due to the characteristic method of synthesis, it is not a uniform silicone polymer. The viscosity assigned to the oil depends on the dominant chain with the specific molecular weight in the mixture. Depending on the manufacturer, a given product may differ in specific viscosity and, therefore, in its properties [13,14]. Unfortunately, during the production process, oils may be contaminated with short-chain polymers, known as low-molecular-weight compounds (LMWCs). Their presence seems to have adverse effects and, despite the purification process, they are difficult to remove. They can cause acute cytotoxicity, deposit in ocular tissues and accelerate the emulsification process as well as promote emulsification of oils with longer chains. Silicone oils are generally characterized by low toxicity and biocompatibility, which is why they are widely used. However, it is suspected that the presence of LMWCs may interfere with SO’s desired properties [15].

### 2.3. Heavy Silicone Oils

Heavy silicone oils (HSOs) are a subgroup of oils that their take name from their higher specific gravity (1.01 g/cm^3^). Due to this feature, compared to the slightly floating classic SOs, they sink in the vitreous chamber. The most desirable are stable HSOs that will not undergo immediate emulsification. For this purpose, mixtures of semifluorinated alkanes (SFAs) with highly viscous PDMS are used, namely Densiron 68 (Fluoron GmbH, Ulm, Germany) and Oxane HD (Bausch & Lomb, Rochester, NY, USA) (with viscosities of 1400 and 3800, respectively) [8]. Beneficial effects are achieved in the indication of complex retinal detachment in the inferior part of the retina, especially with concomitant proliferative vitreoretinopathy, thanks to its unique properties. Unfortunately, detrimental effects on the retina and other ocular tissues do not allow their use as long-term tamponades. More frequent emulsification has been reported with their use than with PDMS alone, reaching up to 65%. Studies show the appearance of emulsification with HSO much faster than with SO—as early as 3 weeks after treatment [16,17]. It is believed that the inflammation induced by HSO induces emulsification, where inflammatory cells behave as surfactants [9]. The advantage of thorough fluid replacement during surgery with the use of perfluorocarbon has been described. An almost complete removal of perfluorocarbon will eliminate the interface between it and SO, reducing emulsification [18].

## 3. Factors Affecting Emulsification

### 3.1. SO Viscosity

This feature depends on the molecular weight and chain length—molecules with a longer chain have a correspondingly higher viscosity. In ophthalmology, oils with viscosity from 1000 to 5000 centistokes (cSt) are commonly used. The onset of SO emulsification is also dependent on oil viscosity. However, there is no clarity regarding the dependence of the degree of emulsification on the viscosity [10,19].

Numerous studies have shown that use of 1000 cSt silicone oil as a tamponade agent is related with earlier emergence of SO emulsification. It should be mentioned, however, that in large number of studies the difference was not statistically significant. Ratanaparkorn et al. [20] noticed that in patients who underwent injection of oil with a lower molecular weight, the emulsification process occurred more frequently than in those who received a higher-molecular-weight oil as a tamponade (63.64% vs. 40%, *p* = 0.08). This phenomenon may be explained by the physical properties of the oil—the higher the viscosity of the oil, the lower the deformation capacity (the higher the energy that is required to disperse) and, as a result, a lower likelihood of the liquid breaking up into smaller droplets. This is consistent with results of a study comparing the use of 1000 and 5000 cSt SO in the treatment of retinal detachment [7,20]. In another study, early removal of the silicone oil was necessary because of the significantly higher frequency of oil emulsification in the 1000 cSt group [21].

However, another study did not show any statistically significant difference in terms of emulsification rate from using higher-viscosity oils (12.1% vs. 12.5%, *p* = 1.000) [22]. Interestingly, not only the type itself but also the brand of oil used can be a factor influencing faster emulsification [1]. Differences between products from various companies may be related to the lack of unified production standards providing equal oil purity [13].

### 3.2. Duration of Tamponade

Time clearly increases the risk of oil emulsification. In the literature, the probable causes of this phenomenon are believed to be the difference between the densities of the fluids inside the eye and the oil, the decrease in the surface tension of the oil over time and the force between silicone oil bubble and aqueous humor during eye movements [10]. How long does it take for oil emulsification to appear? In a small percentage of patients hyperreflective round-shaped droplets were already visible on SD-OCT three months after silicone tamponade. In comparative studies, an interval of 6 months is often used as the cut-off point. The risk of emulsification is higher in patients whose intravitreal oil persists for more than 6 months [1]. It should also be remembered that too short of a period of a tamponade may result in further retinal detachment and the risk/benefit ratio should be considered when scheduling the oil removal procedure. In this regard, a group of patients at high risk of retinal redetachment was examined while maintaining oil endotamponade for as long as possible. The average time after which the features of emulsification appeared was 13.2 ± 4.8 months. Interestingly, in 2 out of 32 cases after 2 years there was no trace of precipitated oil drops [23]. This report contradicts an older study, where out of 150 eyes, all of them experienced emulsification after 12 months [24].

### 3.3. Lens Status

The presence and type of intraocular lens seem to have an influence on the degree of oil emulsification. In a study comparing the presence of natural versus artificial lenses, pseudophakia was associated with more frequent emulsification over time (80% of pseudophakic patients compared to 19% of phakic patients) [25]. In addition to the increased frequency, the amount of emulsified oil is also significantly greater in patients after lens replacement (0.304 mm vs. 0.922 mm—UBM) [26]. In another study, examining risk factors for IOP growth after SO injection, it was noted that pseudophakic patients, in addition to a greater risk of postoperative pressure increase, were more likely to have more oil droplets in the anterior chamber than patients without cataract surgery in the past (*p* = 0.024) [6]. Furthermore, it was noted that combined phacoemulsification with par plana vitrectomy and SO injection correlates with higher SO emulsification grade (*p* = 0.006). This may be related to the greater postoperative inflammatory reaction [27]. Aphakia seems to have the greatest impact on stimulating the emulsification process compared to the presence of a lens (both natural and artificial ones). It is a risk factor for both a higher incidence of emulsification and its degree, as judged by examination of the anterior chamber with ultrasound or on gonioscopy. This is due to the lack of a mechanical barrier—a lens which, when present, prevents the flow of large amounts of oil into the anterior chamber. More importantly, the presence of emulsified droplets does not always appear simultaneously with an increase in pressure. However, there is a correlation between the occurrence of high IOP and the amount of silicone oil in the anterior chamber [26].

### 3.4. Volume of Tamponade

An experimental model was created to find out how the silicone oil tamponade volume influenced the presence of emulsification. A 75% SO-filled chamber was compared with 90%, and this showed that the degree of emulsification was reduced with the larger SO volume. It is hypothesized that when the chamber is filled more completely, the volume of aqueous humor in the chamber is smaller and the relative movement between the two phases (oil and aqueous humor) and their shear force are reduced [28]. A more recent study, also based on an experimental model, partially confirms this thesis. It was noticed that the degree of SO inside of the chamber was significant in reducing shear rate only to a certain extent. The difference was visible when comparing 3 mL volumes with larger values, while comparing only higher volumes with each other did not cause statistically significant differences [29]. Using a scleral buckle at the time of PPV appears to reduce emulsification, probably due to better adhesion of the oil bubble [28].

### 3.5. Nystagmus

Unfortunately, there are few studies on this issue conducted on patients with nystagmus. Most often, research was based on experimental models in which attempts were made to recreate similar conditions prevailing in the vitreous chamber. Considering that in the emulsification process one large bubble turns into smaller ones, physical aspects that may accelerate this should be taken into account. In experimental models, kinetic energy in various forms appears to cause progression of emulsification. This is consistent with the principle that emulsions are generally unstable and are maintained by additional energy. In an analysis that focused primarily on measuring the shear rate, adding a higher-molecular-weight oil caused a decrease in speed between the eye chamber and the oil. Translating these results into the development of emulsification, it was hypothesized that the decrease in velocity reduces the dispersion of oil droplets [30]. In cases of nystagmus, when the eyeballs make repetitive, rhythmic movements, the environment in the oil-filled chamber changes. Repetitive movements increase the shearing force between SO molecules. It is believed that the shear forces described above influence the relationship between nystagmus and emulsification. Yilmaz and Güler in their research demonstrated that in patients with nystagmus, emulsification to varying degrees began much faster. In all eight patients, emulsification occurred within 3 months. Moreover, it led to complications such as open-angle glaucoma both during tamponade with 1000 cs of oil and after its removal. In addition, premature precipitation of the oil droplets forced the SO removal procedure before the projected date, which carried the risk of failure to reach the surgical goal [31].

### 3.6. Others

Longer axial eye length appears to be positively correlated with emulsification. The reason is not entirely clear, but it is probably associated with the mechanism related to the appropriate volume of tamponade filling. Highly myopic eyes also tend to be associated with increased emulsification, perhaps due to weak lens ligaments, reducing the barrier to the forward passage of oil drops [27].

**Figure 1:**
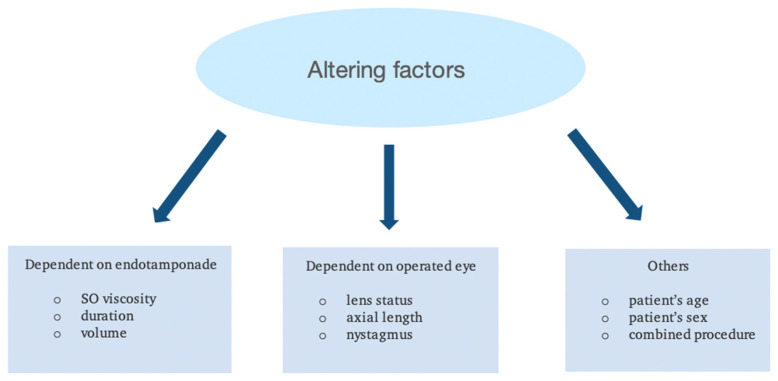
Factors affecting the emulsification process.

Factors that on first glance appear unrelated to the eye can be involved in emulsification. Some researchers have reported that young age may intensify this process [27,32]. Increased eye movements, which stimulate the emulsification process, and aqueous proteins in young people may be the reason for this pattern [30]. It should be noted that this issue is contested [33]. The correlation between gender and onset of oil emulsification seems to be controversial. It has been noticed that men are associated with a higher grade of this phenomenon. The explanation is based on the assumption that they are more active [27]. Other researchers, however, contradicted the significance of the gender factor [1] (Figure 1).

## 4. Complications of Emulsification

### 4.1. Epiretinal Membrane

Epiretinal membrane is a common retinal condition that most often develops idiopathically, but it may also occur secondary to other ophthalmological diseases. The pathophysiology involves cellular proliferation and metaplasia, which are stimulated by inflammatory mediators. Glial or RPE cells may pass into the retina through defects in the internal limiting membrane. They differentiate into myofibroblastic retinal cells, forming the ERM [34,35]. Considering the inflammatory component of this phenomenon, hypotheses arise that the presence of oil may stimulate this process. To date, there is little research describing the influence of oil emulsification on the morphology of the retina and especially on the silicone–retina interface. Recently, hyperreflective areas (that did not correspond to emulsified oil) were noticed in the aforementioned interface in spectral domain optical coherence tomography (SD OCT) images of patients with intraocular silicone. There was a more precisely conglomerated coarse material in the silicone–retina interface and some hyperreflective changes beneath it. Subsequent histopathological analysis of this intraoperatively obtained material revealed macrophages, fibrotic tissue and (not detected in previous OCT images) silicone vacuoles. Despite the removal of this material, over a one-year follow-up, two out of eleven patients developed a preretinal membrane in the location of the described findings. Taking into consideration the inflammatory basis of the novel above-mentioned material and the simultaneous emulsification of oil in patients who underwent vitrectomy with silicone tamponade, a complication in the form of an epiretinal membrane is presumed. More research is needed to thoroughly explore this topic [2].

### 4.2. Keratopathy

Keratopathy is the most common corneal complication in patients who have had vitrectomy with oil injection. The incidence of this pathology ranges from a couple to over a dozen percent, but there are also reports of an even more frequent occurrence [36,37,38,39]. With the use of higher viscosity, the incidence increases (comparatively, 7.0% in the 1000 cSt group vs. 11.9% in the 5000 cSt group) [40]. Moreover, aphakic patients and those with oil in the anterior chamber are at greater risk of developing keratopathy [36,37]. The pathomechanism of this complication may result from a mechanical blockage formed by emulsified oil, which in turn reduces fluid flow across the cornea. The nutritional metabolism of the endothelium, which is normally supplied by the aqueous humor in the anterior chamber, is disturbed [41].

Other adverse effects of emulsified oil on the cornea include damage to the endothelial cell layer and depositions in the stroma. It is speculated that prolonged contact of oil drops with the corneal endothelium has a toxic effect on it, causing progressive loss of its cells [42]. A way to protect the corneal endothelium is the placement of retention sutures in cases of combined iris–lens–retina injury due to ocular trauma. This applies to cases of aphakia and aniridia, where the natural barrier is restored in this way. In studies on a small group of patients, it was noted that the oil tamponade given at vitrectomy, thanks to the presence of retention sutures, usually remained in the posterior chamber without touching the cornea and protected against possible keratopathy [43,44].

### 4.3. Glaucoma and Increase in Intraocular Pressure

The presence of silicone oil can increase intraocular pressure (IOP), leading to glaucoma. We can distinguish several mechanisms leading to this phenomenon, including migration of SO into the anterior chamber, clogging of the trabeculum, silicone oil overfill or pupillary block. The incidence of this complication varies greatly, from 11% [1] to up to half of cases with intravitreal silicone oil [6]. The increase in IOP in the first few days after surgery is not related to rapid emulsification but to other mechanisms, such as too much oil administered [45]. There is little documented association between the increase in pressure and emulsification itself. We have tried to narrow down the above connection only to emulsification.

Firstly, it is worth noting that a bigger quantity of emulsified silicone oil is found in patients with ocular hypertension or those taking antiglaucoma drugs [26,27]. The incidence of glaucoma with emulsification present in the angle was not very common [46]. In a study examining the impact of lens status on pressure values, it was noted that the group of patients with more frequent emulsification was accompanied by an increased incidence of elevated IOP. Given that the difference was statistically significant only after several weeks, the influence on IOP disturbances was suspected to be a result of chronic reaction connected to oil emulsification [25]. This is consistent with the mechanism of emulsification—it occurs over time and is therefore not responsible for sudden increases in intraocular pressure after surgery. Pressure complications related to emulsification occur by the mechanism of an inflammatory reaction or direct infiltration of the angle tissues. Trabecular meshwork is one of the places where dispersed oil droplets accumulate. It is suspected that the presence of oil can initiate the inflammatory process there. The local inflammatory response is mainly mediated by macrophages, no direct lymphocyte activity has been discovered. The attracted macrophages clog the trabeculae, blocking the flow of aqueous humor [47].

A more common increase in pressure after silicone oil injection was observed in the case of lower-viscosity oils. However, the difference was not statistically significant (*p* = 0.096) [6]. This can be the explained by more frequent emulsification of oil in this group compared to oils with higher viscosities. Previous research seems to confirm this thesis [7].

### 4.4. Proliferation Formation

It was noticed some time ago that perisilicone proliferation may occur in the presence of oil drops. In more than half of the patients who underwent pars plana vitrectomy for retinal detachment and proliferative vitreoretinopathy (PVR), proliferations with the presence of emulsified oil returned. By a known mechanism, this adverse effect may lead to retinal detachment. Oil droplets were present on microscopic examination of the preretinal membranes. The findings led to the conclusion that the use of oil for tamponade in patients with recurrent PVR results in more frequent periretinal proliferations. However, it was not specified how much importance the oil emulsification itself may have had in this process, compared to the importance of the postoperative inflammatory process [48]. In a study of patients with PVR, the levels of fibrogenic cytokines and total proteins in retrosilicone fluid were significantly higher compared to the patients without PVR. Therefore, perisilicone proliferation is believed to appear as a consequence of a more intense inflammatory response [49].

### 4.5. Migration of Emulsified SO into the Brain

The presence of SO intracranially is a rare but serious complication of the use of SO endotamponade. Oil emulsification in the cerebral ventricle can be visualized as hyperintensity on T1-weighted magnetic resonance (MR). To confirm the finding, silicone selective imaging and proton MR spectroscopy (MRS) are used [50]. The diagnosis is made with caution because migrated silicone oil can mimic intracerebral hemorrhage [51,52]. The mechanism of SO displacement was not initially clear. Active intracranial migration of SO by the perioptic subarachnoid space of the optic nerve has been recorded, which confirmed one of the earlier hypotheses [52,53]. Unfortunately, the frequency of this complication has not been determined. An attempt was made to determine the incidence of extraocular SO in the orbit, in the optic nerve or in the cerebral ventricles. No such patients were found, which suggests a potentially rare occurrence of SO intracranial migration. However, it was a study on a small group of patients [54]. Most patients with intraventricular SO are asymptomatic, but some may experience headache, nausea and vomiting despite symptomatic treatment. There is no agreement on the removal of intracranial oil. However, excessive intracranial pressure complicated by unremitting symptoms, due to SO within the cerebral ventricles, may require a ventriculoperitoneal shunt procedure [50,52,55].

### 4.6. Vision Loss

Some researchers describe visual deterioration due to emulsification. Droplets settling in the anterior chamber disturb the optical centers of the eyeball. Especially in advanced stages, patients complain of glare and blurred vision [56]. Moreover, decreased vision can be related to the deposition of oil drops in other optical tissues, e.g., on the optic nerve or retina [57]. In addition, other complications mentioned earlier, such as glaucoma or keratopathy, inevitably lead to a gradual decline in visual acuity. In a sense, it is a consequence of other complications [9].

## 5. Evaluation and Diagnostic Methods

The emulsification process is most noticeable in the anterior segment of the eye. If the process is severe, the so-called hyperleon appears in the anterior chamber of the eye. Because it is lighter than the aqueous humor, it collects in the upper part of the AC. SO bubbles may also be located subconjunctivally or infiltrate the corneal endothelium. For the most precise assessment of corneal emulsification, the best tool is a confocal microscope. With this, it is possible to determine oil foci in specific layers of the cornea [58]. Silicone oil droplets have also been described as having a ‘beaded’ arrangement between the implanted lens and posterior capsule [56]. Emulsification can also affect the retina, which can be detected by OCT scans. Histopathologic and experimental research has demonstrated that oil droplets accumulate in almost all structures of the eyeball. The presence of oil emulsification along the optic nerve and in the brain may be visualized by CT and MR scans with special dedicated sequences [50,52,54].

**Figure 2:**
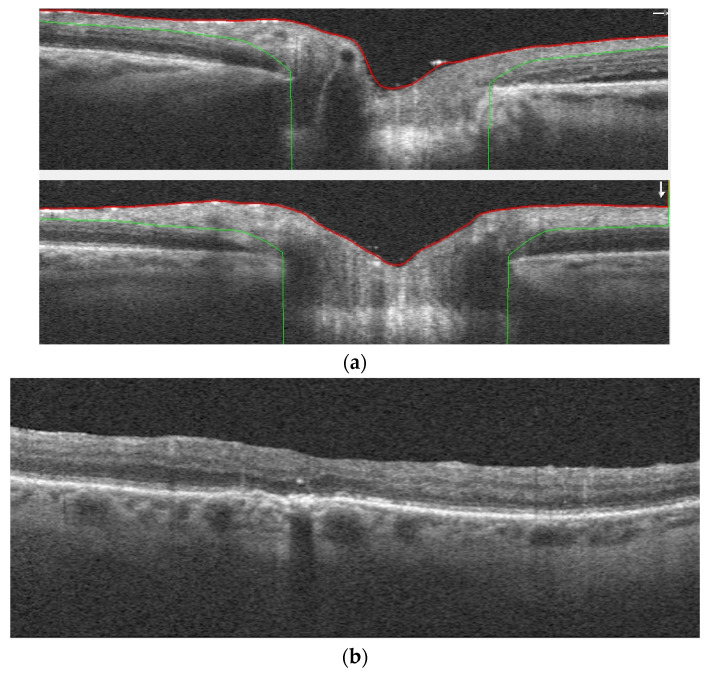
(**a**) OCT image presents hyperreflective droplets which correspond to silicone oil emulsification above the optic disc excavation. (**b**) OCT image presents hyperreflective droplets which correspond to intraretinal silicone oil emulsification.

Thanks to newer diagnostic methods, much attention has been devoted to determining the deposition of oil drops in individual layers of the retina. In patients with oil tamponade after rhegmatogenous retinal detachment, the rate of emulsification detected by OCT was 6% [2]. In cases of oil tamponade, SD OCT can reveal hyperreflective droplets, probably corresponding to emulsified oil (Figure 2a,b). Some drops can also be present after the oil removal procedure. The detected changes were located intraretinally, subretinally or under epiretinal membranes. Similar sites of probable periretinal emulsification were found in patients after the oil removal process. Additionally, intravitreal findings were also identified [59]. Similar hyperreflective vacuoles were also visible in the scans above the optic nerve [60]. Defects in the structure of the internal limiting membrane may contribute to the presence of emulsified oil fragments in the inner layers of the retina. Thus, the use of oil in macular hole surgery may result in poorer postoperative outcomes [61]. As we have already noted, ERMs sometimes have a greater affinity to form in the presence of silicone oil. When comparing idiopathic preretinal membranes with ERMs formed after SO injection, morphological differences can be noticeable in OCT imaging. In SO preretinal membranes, the fovea centralis is statistically thicker (median 104.5 µm vs. 17.3 µm), as well as the CRT (587 µm vs. 487 µm). These differences are probably due to its double layer, where the upper one corresponds in histopathological examination to a granulomatous part with emulsified oil [62]. Retinal edema in the areas of emulsification may also be seen in the scans. Cystic spaces have a presumably inflammatory origin or result from long-standing retinal detachment [59,62].

## 6. Conclusions

Because it is transparent and provides a long-term tamponadę, SO still plays a key role in vitreoretinal surgery. To avoid complications and eliminate the side effects of oil tamponade, it is usually removed after a few months. However, it is possible to reduce the incidence SO emulsification. Regarding the choice of oils, adding a few heavier chains to new lower-viscosity oils seems to reduce the emulsification, achieving results similar to 5000 cSt oils. The use of these novel modified oils may make it possible to discontinue the use of 5000 cSt oils in the future [63]. OCT can be used to diagnose emulsification and monitor the condition of the patient’s retina.
